# Regulatory T cells control strain specific resistance to Experimental Autoimmune Prostatitis

**DOI:** 10.1038/srep33097

**Published:** 2016-09-14

**Authors:** Maria L. Breser, Andreia C. Lino, Ruben D. Motrich, Gloria J. Godoy, Jocelyne Demengeot, Virginia E. Rivero

**Affiliations:** 1 Centro de Investigaciones en Bioquímica Clínica e Inmunología (CIBICI-CONICET), Departamento de Bioquímica Clínica, Facultad de Ciencias Químicas, Universidad Nacional de Córdoba, Haya de la Torre y Medina Allende, Ciudad Universitaria, 5016, Córdoba, Argentina; 2 Instituto Gulbenkian de Ciência, Oeiras, Portugal

## Abstract

Susceptibility to autoimmune diseases results from the encounter of a complex and long evolved genetic context with a no less complex and changing environment. Major actors in maintaining health are regulatory T cells (Treg) that primarily dampen a large subset of autoreactive lymphocytes escaping thymic negative selection. Here, we directly asked whether Treg participate in defining susceptibility and resistance to Experimental Autoimmune Prostatitis (EAP). We analyzed three common laboratory strains of mice presenting with different susceptibility to autoimmune prostatitis upon immunization with prostate proteins. The NOD, the C57BL/6 and the BALB/c mice that can be classified along a disease score ranging from severe, mild and to undetectable, respectively. Upon mild and transient depletion of Treg at the induction phase of EAP, each model showed an increment along this score, most remarkably with the BALB/c mice switching from a resistant to a susceptible phenotype. We further show that disease associates with the upregulation of CXCR3 expression on effector T cells, a process requiring IFNγ. Together with recent advances on environmental factors affecting Treg, these findings provide a likely cellular and molecular explanation to the recent rise in autoimmune diseases incidence.

Susceptibility or resistance to autoimmune disorder has a clear genetic component in human and in mouse models^1^. However, the multifactorial nature of organ specific autoimmunity has limited our understanding of the biology behind the processes that define which organism is affected, and which organ is targeted upon common immunoregulation disorder^2^. Murine models of autoimmune disease relying on immunization with autoantigens offer the possibility to focus on a defined tissue target and dissect susceptibility factors others than those involved in the original immunological stimuli^3^,^4^.

According to the auto-antigen used as immunogene, mouse strains display specific disease susceptibility or resistance. For instance alpha-myosin or collagen immunization induces experimental autoimmune cardiomyopathy or arthritis, respectively, in BALB/c but not in C57BL/6 mice, while similar immunization with myelin oligodendrocyte glycoprotein leads to encephalomyelitis in the latter but not in the former strain^5^,^6^,^7^. Other models of induced autoimmunity rely on immunization with total protein extract of a given organ, multiplying the number of antigens in a single protocol and thus presumably enlarging the repertoire of effector cells, as is the case for experimental autoimmune prostatitis (EAP) where the immunogene is a protein extract of the whole prostate^8^. In these type of models too, the combination of strain and antigen mixture defines the outcome of immunization, and EAP is evident in NOD and C57BL/6 but undetected in BALB/c mice^9^.

The most commonly evoked notion to explain susceptibility/resistance to organ-specific autoimmunity is that MHC-antigen complexes are heterogenous in number or affinity among different strains^10^. Alternatively, as selection of the TCR repertoire appears to result in the purging of autoreactivity, this process may be more or less efficicient in various strains^11^. However, in most models of immunization with self-protein, antigen specific immune response can be detected whether disease follows or not, indicating that other layers of immune control, beyond the engagement of effector cells, condition the evolution to disease^12^. Intriguingly, the notion that some strains are biased toward specific cytokine profiles, e.g. Th1 for C57BL/6 and Th2 for BALB/c mice, hardly explain the mirror image presented above, i.e. that the same strain is susceptible to some inflammatory diseases and not to others^13^. Together, these observations leave open the possibility that specific control, dampening the progression of immune responses at the site of immunization to organ infiltration and associated tissue destruction, partipates in resisting the pathological process^14^. Among those, suppression by regulatory T cells (Treg) is a possibility, as these cells are now known to exert other functions in addition to their role in preventing effector cell activation^14^,^15^. Null mutation in Foxp3, a transcription factor necessary and required for Treg differentiation and function^16^, unleashes a large repertoire of auto-reactive cells that escape negative selection and lead to a multiorgan autoimmune disease that is fatal in early age in both mice and humans^17^. Treg dampen immune responses in a cell contact or justacrine manner upon TCR engagement, through their expression of immunosuppresor cytokines (TGF-β and IL-10) and check-point molecules (CTLA4)^18^,^19^. They were also recently implicated in tissue-healing and damage control, notably through the expression of amphiregulin^20^.

Over the past decade our laboratory developed a mouse model of EAP^8^,^9^ that serves to mimick chronic prostatitis/chronic pelvic pain syndrome (CP⁄CPPS), one of the most prevalent diseases in urologic clinics^21^. CP⁄CPPS is an inflammatory disease that affects men younger than 50 years old in the absence of detectable infection^22^, and associates with Th1 like prostate specific reactivities^23^. Likewise, immunization of young adult males mice with prostate proteins together with adjuvants leads to prostatitis^8^, a progression strictly dependent on INFγ and the associated chemokine receptor CXCR3^9^,^24^. Although all mice strains mount antigen specific responses upon immunization, NOD mice develop a severe disease, C57BL/6 animals exhibit moderate prostate infiltration and BALB/c mice show no prostate tissue alterations^9^,^25^.

We used the EAP model to assess the role of Treg in defining strain specific susceptibility or resistance to induced autoimmune disease. By treating mice with anti- CD25Ab to transiently deplete Tregs at the time of EAP induction, we reveal that these cells dampen a Th1/Tc1 response and ensuing prostrate infiltration, in all mice, including in the resistant BALB/c strain. These findings indicate that beyond genetic factors imposing strain intrinsic effector cell repertoire or tissue resilience, resistance to EAP is promoted by a regulatory mechanism that dampens a preexisting potential for tissue infiltration and associated destruction. In view of the natural dynamics and plasticity of Treg, these findings strengthen the notion that both genetic and environmental factors contribute to autoimmune disease susceptibility, the latter more easily explaining the recent rise in disease incidence.

## Results

### Both EAP susceptible and resistant mice mount an immune response upon prostate antigen immunization

**Figure 1:**
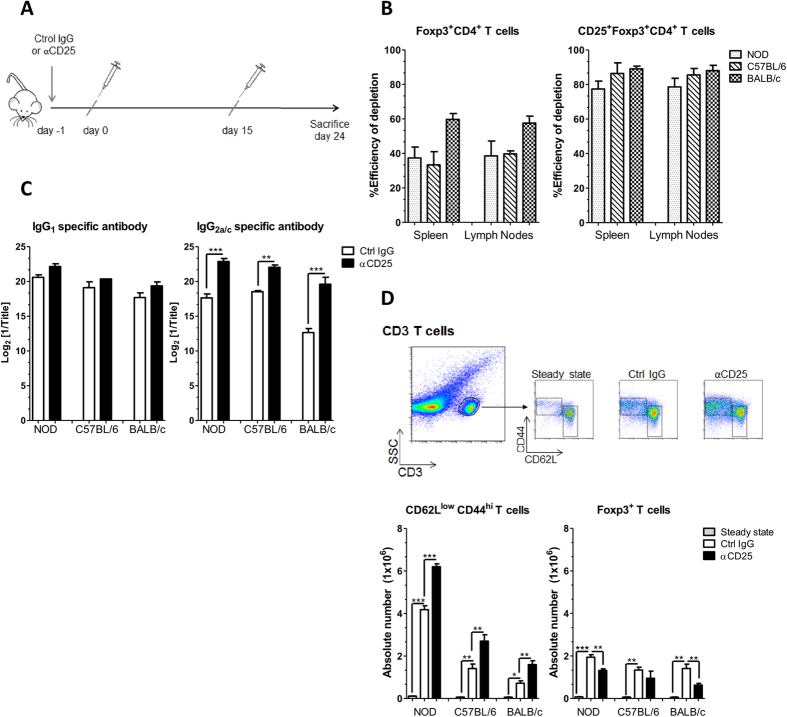
CD25^+^ cell depletion enhances immune responses in all strains. (**A**) Experimental scheme representing the protocol of treatment and immunization: i.v. injection with anti-CD25Ab or control Ab on day −1, i.d. immunization with PAg plus adjuvant on days 0 and 15, and mice sacrifice on day 24. This protocol was applied to six to 8 week old male NOD, C57BL/6 and BALB/c mice. (**B**) Foxp3^+^ CD4^+^ and CD25^+^ Foxp3^+^ CD4^+^ T cell depletion efficiency tested 48 h after anti-CD25 Ab treatment measured by Flow cytometry. (**C**) PSBP-specific IgG1 and IgG2a-c levels measured in serum samples on day 24 by ELISA as described in Materials and Methods. (**D**) Gating strategy used to evaluate absolute numbers of effector (CD62L^low^CD44^hi^) T cells and Treg (CD4^+^ Foxp3^+^) cells in draining LN from individual NOD, C57BL/6 and BALB/c mice in steady state conditions (without treatment) or on day 24 of the experimental protocol. Data are shown as mean ± SEM, n = 6 mice per group and representative of two independent experiments. The *p* values were obtained using one-way ANOVA followed by Bonferroni post-hoc analysis.

To assess whether regulatory T cells naturally limit the induction of EAP in mice we tested three mouse strains presenting varying degree of susceptibility or resistance to the disease. We first choose to compare NOD, C57BL/6 and BALB/c mice that develop severe, mild and no disease, respectively^9^,^25^. Animals were submitted to a single injection of the depleting anti-CD25 Ab (PC61), or of the isotype control rat IgG1 (YCATE), one day before immunization (Fig. 1A). Analysis of Foxp3 expressing cells at steady state confirmed that NOD and BALB/c mice present the lowest and highest frequency of regulatory T cells, respectively^26^,^27^. In all strains Foxp3^+^ cells expressed similar levels of Foxp3 and GITR and about half of them expressed CD25 (Supplementary Fig. 1A,B). Consistently with this phenotype, a single injection of PC61 leads to about 50% reduction in CD4^+^ Foxp3^+^ T cells and remove about 80–90% of CD4^+^ CD25^hi^ Foxp3^+^ T cells in all strains of mice, as tested 48 h post injection (Fig. 1B, Supplementary Fig. 1C). This depletion is transient, and in absence of other interventions normal number of cells is recovered in the following 20 days^28^,^29^. Immunization with prostate proteins (PAg) emulsified in CFA was conducted as reported before^9^ and consisted of a priming injection followed by a boost 15 days later. Animals were analyzed at day 24, a time point our previous studies established as optimal for EAP evaluation^9^,^24^. To assess the amplitude of the immune response we first measured serum Ig specific to prostate steroid-binding protein (PSBP, also named prostatein), an abundant immunogenic antigen from the prostate^30^,^31^. As previously described^9^, all strains mounted a robust humoral response upon immunization, with high titers of antigen specific IgG1 and IgG2a/c. In the three strains, treatment with anti-CD25 Ab readily increased PSBP specific IgG2a/c titers as indicated by several log_2_ differences (Fig. 1C). We next monitored the extent of overall cellular activation by performing FACS analysis of spleen and lymph nodes draining the prostate (Fig. 1D and Supplementary Fig. 1D). Mice were analyzed in steady state conditions or after immunization and anti-CD25 or control Ab treatment. In all strains, immunization *per se* readily increased the number of CD62L^low^CD44^high^ memory/effector T cells recovered when compared to steady state conditions, and prior anti-CD25 Ab administration amplified this effect (Fig. 1D). Similarly, the number of CD62L^high^CD44^high^ central memory T cells recovered upon immunization was increased in mice that were pretreated with anti-CD25 (Supplementary Fig. 1C). In control as in treated groups, the response was higher in NOD than C57BL/6 mice, and BALB/c animals were the less responsive. Strikingly, in all strains, immunization *per se* readily increased the number of CD4^+^ Foxp3^+^ cells when compared to steady state conditions (Fig. 1D). This increment was partially affected by prior anti-CD25 Ab treatment in NOD and BALB/c mice, but not in B6 animals, suggesting strain specific differences in the kinetics of Treg recovery/proliferation (Fig. 1D). Together these results indicate that all 3 strains do mount an immune response, limited by CD25^+^ regulatory cells, upon PAg immunization.

### CD25^+^ regulatory cells dampen a Th1/Tc1 immune responses in both EAP susceptible and resistant mice

**Figure 2:**
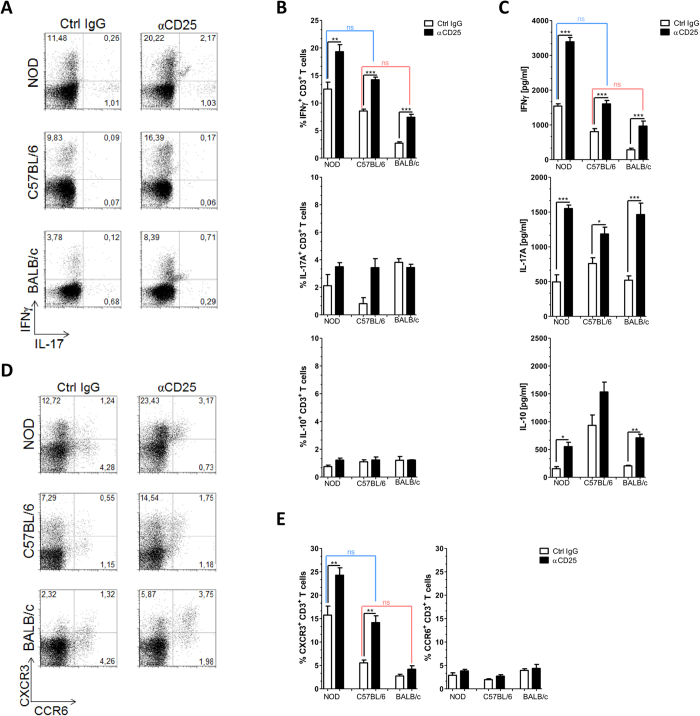
CD25^+^ cell depletion diverts specific immune response towards a Th1/Tc1 pattern. Mice were treated as in Fig. 1, pooled draining LN from individual NOD, C57BL/6 and BALB/c mice were analyzed at day 24, either straight (**A**) or after stimulation with PSBP for 72 h (**B–E**). (**A**) Flow cytometric analysis for IFNγ and IL-17A intracellular expression in CD3^+^ cells isolated from draining LN following *in vitro* stimulation with PMA/inomycin for 5 h in the presence of Golgi Stop. (**B**) Percentage of IFNγ^+^ CD3^+^, IL-17A^+^ CD3^+^ and IL-10^+^ CD3^+^ T lymphocytes isolated from draining LN following *in vitro* stimulation with PSBP for 72 h and then 5 h. PMA/ionomycin incubation in the presence of Golgi Stop as described in Materials and Methods. (**C**) Culture supernatants of mononuclear cells from draining LN isolated on day 24 of the experimental protocol, analyzed by ELISA. (**D**) Representative flow cytometry dot plots of gating strategy and CXCR3 and CCR6 staining on the gated CD3^+^ T cell population after 72 h of PSBP stimulation. (**E**) Percentages of CXCR3^+^ and CCR6^+^ in CD3^+^ T cells after 72 h of PSBP stimulation. Data are shown as mean ± SEM, n = 6 per group, and are representative of two independent experiments. The *p* values were obtained using one-way ANOVA followed by Bonferroni post-hoc analysis; ns, non significant.

We further assessed the class of the immune response under the control of CD25^+^ regulatory cells in the three strains. Intracellular staining for IFNγ and IL-17 in gated CD3^+^ T cells from draining LN cells collected in the experiments described above revealed that anti-CD25 Ab treatment amplified a Th1/Tc1 rather than a Th17 response (Fig. 2A). To focus the analysis on antigen specific T cells, draining LN cells were maintained in culture in conditions favoring survival and expansion of PSBP specific cells^9^. Three days later, both recovered cells and culture supernatants were analyzed by FACS (Fig. 2B) and ELISA (Fig. 2C), respectively. In agreement with previous work indicating that IFNγ is required for EAP^9^,^24^, antigen specific IFNγ^+^CD3^+^ proportion was increased in NOD when compared to C57BL/6 mice and reduced in BALB/c animals. Indeed, FACS analysis of the cells recovered from the cultures, after intracellular staining for IFNγ and IL-17, showed the highest levels of IFNγ^+^CD3^+^ in the NOD strain after anti-CD25Ab treatment (Fig. 2B). Analysis of culture supernatants corroborated the same pattern; anti-CD25 Ab treatment raised the level of antigen specific secretion of IFNγ in all strains, with treated BALB/c mice approaching the values obtained in untreated C57BL/6 animals, and treated C57BL/6 approaching the values obtained in untreated NOD mice (Fig. 2C). In contrast, the antigen specific production of IL-17, while also increased upon anti-CD25 Ab treatment, was not strain specific. While the frequency of IL10^+^CD3^+^ cell did not change, an increased IL-10 secretion (notably in NOD and BALB/c mice) was observed in anti-CD25 Ab treated mice, suggesting that other cells than T cells produced IL-10, and these are also under Treg control. Surface staining for the chemokine receptors CXCR3 and CCR6 respectively associated with Th1/Tc1 and Th17/Tc17 classes of response^32^, confirmed a Th1/Tc1 strain-specific pattern (Fig. 2D,E). Antigen specific CXCR3 T cells were more abundant in NOD than C57BL/6 mice, and less prevalent in BALB/c animals, while CCR6 expression was not correlated to EAP susceptibility. As for IFNγ, CXCR3 analysis indicated that treatment with anti-CD25 Ab promoted a C57BL/6-like response in BALB/c animals and a NOD-like response in C57BL/6 mice (Fig. 2D,E).

### CD25^+^ regulatory cells prevent Prostatitis in susceptible and resistant mice

**Figure 3:**
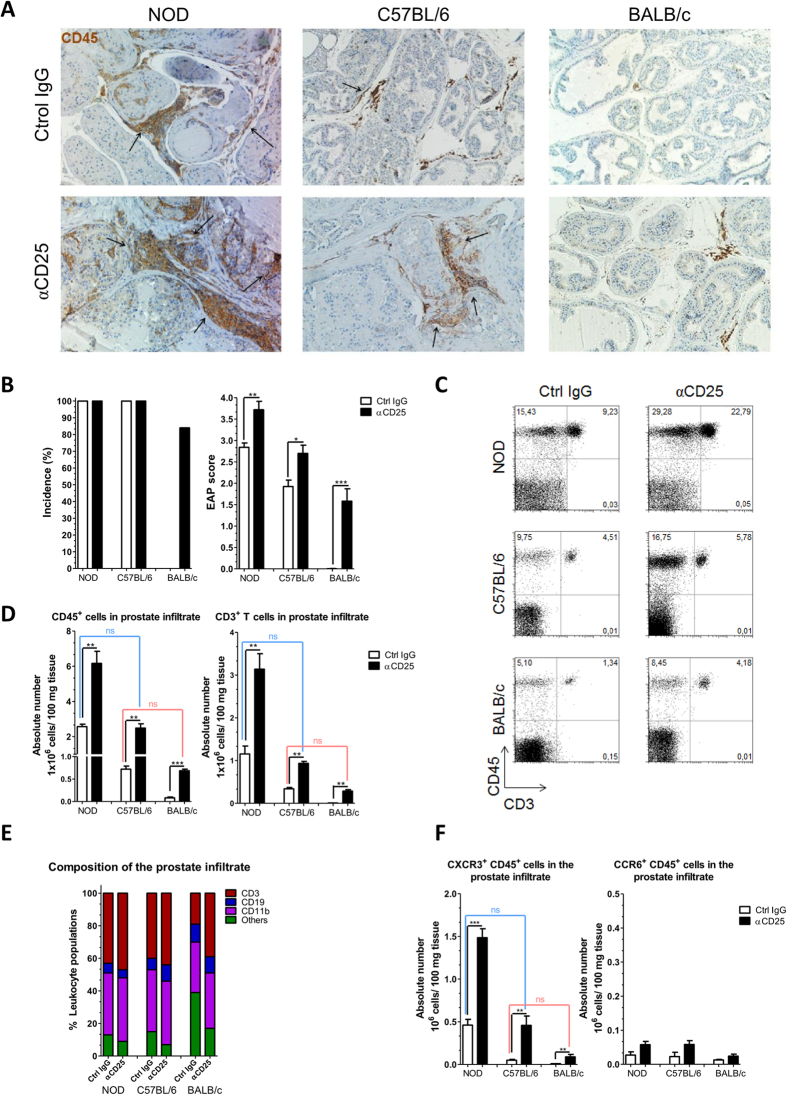
CD25 cell depletion promotes Experimental Autoimmune Prostatitis. NOD, C57BL/6 and BALB/c mice were treated as in Fig. 1. (**A**) Representative immunohistochemistry assays for CD45^+^ cells in prostate tissue sections from NOD, C57BL/6 and BALB/c mice analyzed at day 24. Arrows indicate the CD45^+^ cell clusters infiltrating the prostate gland. Original magnification X200. (**B**) Incidence and prostate histological scores from mice under study. (**C**) Presence of leukocytes infiltrating the prostate gland from animals under study, representative dot plots CD45^+^ versus CD3^+^ cells. (**D**) Bar graphs shown absolute numbers of CD45^+^ and CD3^+^ cells infiltrating the prostate gland from animals under study. (**E**) Percentages of CD3^+^, CD19^+^, CD11b^+^ and others in the prostate infiltrate from animals under study. (**F**) Absolute number of CXCR3^+^ CD45^+^ and CCR6^+^ CD45^+^ cells infiltrating the prostate gland from mice under study. Data are shown as mean ± SEM, n = 6 mice per group, and are representative of two independent experiments. The *p* values were obtained using one-way ANOVA followed by Bonferroni post-hoc analysis; ns, non significant.

Prostatitis is clearly evidenced by immunohistochemistry on prostate tissue to reveal CD45^+^ leucocytes infiltration and to evaluate tissue damage (Fig. 3A,B). Immunized control mice confirmed the high prevalence and severity of EAP in NOD mice, the milder manifestation in C57BL/6 animals and the absence of infiltrates in BALB/c mice. Treatment with anti-CD25 Ab at the induction phase enhanced tissue cell infiltration and destruction in both susceptible strains. Strikingly, the treatment promoted disease in the otherwise resistant BALB/c mice, with an incidence of 5 out of 6 tested animals, and a severity comparable to that of control C57BL/6 animals (Fig. 3B). Significant histological alterations were detected in prostate tissue from anti-CD25 Ab treated NOD mice consisting in diffuse perivascular and stromal mononuclear cell infiltration accompanied by edema and severe tissue disorganization. Prostate samples from anti- CD25 Ab treated C57BL/6 mice exhibited multifocal perivascular and stromal mononuclear cell infiltration. Finally, significant histological alterations were detected in prostate tissue from anti-CD25Ab treated BALB/c mice, consisting in focal perivascular and stromal mononuclear cell infiltration, accompanied by edema (Fig. 3A).

We next quantified and characterized prostate infiltrating leukocytes by performing FACS analysis (Fig. 3C–F and Supplementary Fig. 2A). In a remarkable concordance with the histological scores, the number of leucocytes (CD45^+^) or T cells (CD3^+^) recovered from prostate tissues were more elevated in NOD mice than in C57BL/6 animals (about 2 folds), and BALB/c presented with the lowest values. For these parameters too, administration of anti-CD25 Ab at the induction phase of EAP increased the values, such that treated BALB/c mice resembled control C57BL/6 animals and treated C57BL/6 mice resembled untreated NOD animals. When analyzing the leukocyte populations composing the infiltrate, CD3^+^ and CD11b^+^ cells were the most abundant while the frequency of CD19^+^ cells was low. These proportions were similar irrespective of anti-CD25 Ab treatment, with the exception of untreated healthy BALB/c mice which as expected exhibited lower frequency of CD3^+^ cells within the infiltrates (Fig. 3E). Inside the CD3^+^ cellular subset about 60% were CD4^+^ and 40% were CD8^+^ T cells, irrespectively of the strain or the treatments (Supplementary Fig. 2A). In agreement with a major role for CXCR3 in promoting T cell migration from the lymph nodes to the tissue, the number of infiltrating T cells expressing this chemokine receptor positively correlated with disease severity (Fig. 3F). Contrarily to our analysis of the draining lymph nodes (Fig. 2D), the frequencies of T cells expressing CXCR3 in the infiltrates were constant across the different experimental groups (Supplemental Fig. 2B). These results support the notion that CXCR3 expression is acquired in the lymph nodes and facilitates migration to the tissue. Finally, little or no CCR6^+^ cells were detected in prostate infiltrates (Fig. 3F, Supplementary Fig. 2B). Together, these results indicate that CD25^+^ regulatory cells dampen a tissue specific Th1/Tc1 deleterious autoimmune response, triggered by immunization, in both EAP susceptible and resistant strains. In turn, this finding indicates that EAP resistance in BALB/c mice is not solely the result of efficient negative selection of the TCR autoimmune repertoire, or of poor antigen presentation.

### CD25^+^regulatory cells dampen CXCR3 expression on effector T cells, a process requiring IFNγ

**Figure 4:**
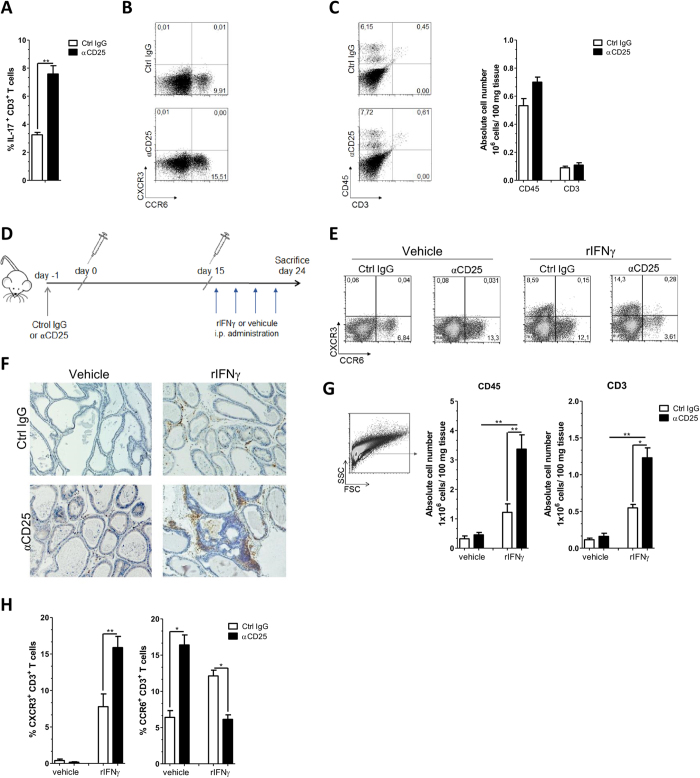
CD25^+^ cell depletion enhances IFNγ dependent CXCR3 T cell expression and thus autoimmune prostatitis in *NOD-IFN*γ ^−/−^ mice. Six weeks old NOD-IFNγ^−/−^ mice were treated as in Fig. 1A and their draining LN cells and prostate infiltrates were analyzed at day 24. Draining LN cells from individual mice were stimulated with PSBP for 72 h. (**A**) Percentages of IL-17A^+^ cells in gated CD3^+^ lymphocytes in draining LN. (**B**) Representative dot plot analysis of CXCR3 and CCR6 staining in gated CD3^+^ T cell from draining LN. (**C**) Representative dot plot analysis and bar graphs showing absolute numbers of CD45^+^ and CD3^+^ cells infiltrating the prostate gland from NOD-IFNγ^−/−^ mice analyzed at day 24. (**D**) Experimental scheme representing the protocol of anti-CD25 Ab treatment, immunization, i.p. treatment with rIFNγ on day 16, 18, 20 and 22 and mice sacrifice on day 24. (**E**) Representative flow cytometry dot plots of CXCR3 and CCR6 staining in gated CD3^+^ T cell population from draining LN. (**F**) Representative immunohistochemistry assay for CD45^+^ cells in prostate tissue sections from control or anti-CD25 treated NOD-IFNγ^−/−^ mice injected with vehicle or rIFNγ. (**G**) Presence of leukocytes infiltrating the prostate gland from anti-CD25 treated NOD-IFNγ^−/−^ mice treated with rIFNγ or vehicle evaluated by flow cytometry. Bars show absolute numbers of CD45^+^ and CD3^+^ cells infiltrating the prostate gland from animals under study. (**H**) Percentages of CXCR3^+^ CD3^+^ and CCR6^+^ CD3^+^ T cells infiltrating in the prostate gland from NOD-IFNγ^−/−^ mice under study. Data are shown as mean ± SEM, n = 4 mice per group, and are representative of three independent experiments. The *p* values were obtained using one-way ANOVA followed by Bonferroni post-hoc analysis.

In a previous work, we evidenced that IFNγ is required for EAP occurrence through its essential role in the induction of CXCR3 expression by T cells, a mechanism of trans-regulation^9^. As anti-CD25 Ab treatment promoted both IFNγ and CXCR3 expression upon immunization, we next asked whether CD25^+^ regulatory T cells affected CXCR3 expression independently of their role in dampening cytokine production. We analyzed NOD-IFNγ^−/−^ mice that are fully resistant to EAP^9^. These mice mount a Th17/Tc17 type of response upon immunization as indicated by the presence of IL-17 producing and CCR6 expressing T cells in pooled draining LN at day 24 (Fig. 4A,B). Both T cell phenotypes were amplified by anti-CD25 Ab administration before immunization, while CXCR3 expression remained undetectable (Fig. 4A,B). In addition, NOD-IFNγ^−/−^ treated with anti-CD25 Ab remained resistant to EAP showing no infiltration or damage in the prostate gland (Fig. 4C). To allow for CXCR3 expression, we complemented the IFNγ deficiency by providing recombinant-IFNγ (rIFNγ) through repeated injection during the boosting phase of the immunization^9^ (Fig. 4D). This treatment *per se* promotes CXCR3 expression and increases the frequency of CXCR3^+^ cells in draining LN^9^. In the control conditions (vehicle only), and as observed above, anti-CD25 Ab treatment increased the frequency of cells expressing CCR6. In contrast, in presence of rIFNγ, anti-CD25 Ab treatment increased by twofold the frequency of antigen specific CXCR3^+^ cells, an increment associated with a twofold reduction in the frequency of CCR6^+^ cells (Fig. 4E). This switch in the class of chemokine receptors expressed by T cells upon immunization, and its amplification by the depletion of CD25^+^ regulatory T cells, resulted in prostate infiltrations with evident tissue destruction (Fig. 4F), to an extent similar to that observed in WT animals (see Fig. 3A), and with a disease score close to 3. In the same animals, cellular analysis of prostate infiltrates revealed increased number of leucocytes and T cells (Fig. 4H), reaching numbers similar to those observed in WT mice (see Fig. 3D). From these data, we conclude that CD25^+^ regulatory cells dampen the differentiation of CXCR3^+^ cells, by a process independent of effector T cell autocrine IFNγ secretion.

## Discussion

By performing limited and transient Treg depletion at the time of EAP induction in susceptible and resistant mice we reveal that, beyond strain specific repertoire or tissue resilience, a layer of immune control dampens pathological responses, to the point of conferring disease resistance to BALB/c mice. This approach applied to mutant mice also provided key information regarding the role of specific inflammatory cytokines and chemokine receptors in EAP onset. Together, this work provides new clues to our understanding of susceptibility to autoimmune disease and its variation in complex genetic and environmental settings, as it is the case for humans.

To assess the role of Treg in defining the susceptibility and severity to EAP we choose a loss of function approach and resorted to anti−CD25 antibody mediated cellular depletion. The CD25 molecule is expressed by most Foxp3^+^ regulatory T cells, especially those functionally engaged in regulation^33^. It is also expressed by activated T cells, albeit transiently, a small subset of B cells, and few dendritic cells^34^,^35^. While Ab mediated depletion may lack in specificity when compared to targeted genetic engineering, it offers the unique opportunity to test various genetic background in a short time. Moreover, available genetic models were not directly adapted to serve our objectives. Mice bearing a null mutation in the Foxp3 gene are fully deficient in Treg and develop spontaneously a severe and multi-organ pathology leading to early death^36^, a model not easily amenable for strain specific analysis and not adapted for the case of prostatitis as death occurs before full development of the prostate (unpublished observation). Animals bearing the diphtheria toxin receptor gene under the transcriptional control of Foxp3 allow for Treg depletion in adults, however discrepancy of results across the available models^37^,^38^ still await disambiguation before engaging in organ and strain specific analyses. Moreover, diphtheria toxin has side effects in mice, and more specifically, its toxicity is increased when combined with adjuvants like CFA^39^. Administration of a single dose of the monoclonal anti-CD25 Ab PC61 in mice, induces a depletion of CD25 expressing cells that is transient, due to Ab clearance and homeostatic rebound^28^, and cannot be prolonged due to the Ab intrinsic immunity in mice (unpublished observations). In addition to cellular depletion, PC61 may functionally inactivate the remaining Treg through antibody-mediated blockade of IL-2 signaling^40^,^41^. However, depletion and functional inactivation remain moderate as no manifestation of systemic or tissue specific autoimmunity has ever been reported or noted^42^ (and unpublished observation) upon a single injection of PC61.

We found that a reduction in regulatory cell number during the priming phase of EAP was enough to break down self-tolerance in the resistant BALB/c strain and worsen disease severity in both the mildly and the highly susceptible C57BL/6 and NOD mice, respectively. A role for Treg in preventing spontaneous prostatitis occurrence in susceptible mice has been evidenced earlier. Neonatal thymectomy in mice results in spontaneous autoimmunity targeting varying organs, including the prostate^43^. Several line of evidences indicate that disease in this system results from alteration in Treg repertoire and function, and specifically, adoptive transfer shortly after day 3 thymectomy of Treg isolated from healthy donor prevents EAP^44^. In our system, effector and potentially pathogenic cells are primed and boosted by the immunization, and transient Treg depletion evidence that negative selection does not explain disease resistance in BALB/c mice. As acknowledged above anti CD25 Ab targets other cells than Treg. In a model of induced Autoimmune Experimental Uveitis, C57BL/6 mice treated with anti CD25 Ab showed decreased numbers of Treg but also γδ T cells and CD11c^+^ CD3^−^CD25^+^ dendritic cells that could explain the observed reduced Th17 and enhanced autoreactive Th1 response^35^. In our study we did not discriminate the relative contribution of Treg and other CD25 expressing cells in defining EAP susceptibility.While additional layers of regulation may well take place, our results establish that susceptibility and resistance to EAP is defined by dominant regulatory mechanisms and not merely by recessive tolerance.

Previous studies have reported that Treg depletion may converts specific strains from a non-responder to a responder phenotype in infectious setting (e.g. ref. 45). In setting of autoimmunity, most studies used Treg depletion to evidence their protective role in animals prone to spontaneously develop disease. This is the case of a recent work revealing that transient Treg depletion induced by administration of a low dose and very short regimen of diphtheria toxin in Foxp3-DTR mice induces disease in BALB/c CrSlc, a substrain well know for its susceptibility to gastritis^46^. In our experimental setting of induced autoimmunity, immunization provides for normalization of the priming event, across strains. With this approach, the role of regulatory cells in susceptibility or resistance to disease can be addressed beyond questioning, or eluding, the still obscure issue of the original tissue-antigen specific priming of pathogenic cells.

Despite Treg depletion prior to immunization, BALB/c mice developed a moderate immune response and a mild disease, comparable to those occurring in untreated C57BL/6 animals. As previously reported, we found higher frequencies of CD4^+^ Foxp3^+^ cells in peripheral lymphoid organs of BALB/c mice when compared with NOD and C57BL/6 animals, in steady state conditions^26^,^27^. Moreover, BALB/c when compared to C57BL/6 Treg were shown to be more efficient at suppressing conventional cell^26^, a result not reproduced later^27^. It is conceivable that our partial depletion preserved antigen specific Treg in BALB/c more than in C57BL/6 mice, due to natural enrichment or higher efficiency in the former strain. Alternatively, BALB/c mice could carry less antigen specific conventional cells. The two notions seems actually related, as indicated by recent works confirming that the repertoire of self-reactive conventional and regulatory T cells is sorted according to specific properties of the interactions between the TCR and MHC-self-peptide in the thymus^47^,^48^,^49^, and it is easily conceivable that these properties are tuned by specific MHC alleles.

Our results evidence that in all strains disease severity associates with enhanced generation of antigen specific T cells expressing CXCR3, but not CCR6. In a previous work we showed that IFN-γ is required for EAP occurrence due to its specific role in promoting effector T cells migration to the prostate tissues^9^. This study also revealed that IFNγ acts in *trans* to upregulate CXCR3 and downregulate CCR6 chemokine receptors on effector T cells. In turn, this work indicated that irrespectively of whether effectors cells are IFNγ or IL-17 producers, it is their chemokine receptor expression pattern that determines their capacity to infiltrate the tissues. Here, in the three mouse strains under study, we show that a transient depletion of Treg cells at the inductive phase of the disease promotes a Th1/Tc1 response, with moderate effect on the Th17/Tc17 subset. In contrast, in mice deficient for IFNγ, the same treatment enhanced a robust Th17 response, although not associated with EAP. These results are consistent with the widely accepted notion that Treg inhibit a large range of immune cells, including Th1, Th17, Th2 and also CD8^+^ T cells. In our system, the context is imposed by CFA that provides the signal necessary to define the class of responses. In turn, our results support the notion that in wild type animals and under inflammation promoted by CFA, Treg dampen the dominant IFNγ responses even in the Th2 biased BALB/c background.

Th1 and Th17 pathogenic effectors cells once induced in the periphery must traffic to their target organs and this traffic is associated with the expression of specific chemokine receptors and also with the induction of appropriate chemokines in the target organ^50^. More relevant to the mechanism of autoimmune disease, our results also support the notion that the pathology results from the upregulation of CXCR3 on effector cells, rather than on specific cytokine profiles. This is best illustrated in our analysis of IFNγ deficient mice, where although effector cell numbers are increased through transient Treg depletion, these are pathologic only if exposed to IFNγ. In turn these results indicate that BALB/c mice are resistant to EAP, not because of a lack of potential to mount an antigen specific response, but owing to the inhibition of Th1/Tc1 response by Treg, that prevent the production of IFNγ required for effector cells migration to the prostate.

Choosing three strains to cover a spectrum of susceptibility, from the NOD with exacerbated disease, the C57BL/6 with mild inflammation and to the resistant BALB/c mice, we show that transient Treg depletion moves each strain to the next degree of severity: the severity of prostate infiltration in treated BALB/c animal is similar to that of untreated C57BL/6 and likewise treated C57BL/6 animals appear as affected as untreated NOD mice (Fig. 3). This finding is striking as it indicates that mild disturbances in the Treg pool can confer susceptibility to a resistant strain, offering in turn a likely explanation to the recent rise in autoimmune disease incidence in developed countries^51^. This increment was too rapid to result from enrichment of the gene pool with susceptibility alleles. Along the hygiene hypothesis, societal factors such as systematic vaccination reduced the occurrence of repeated immune activation that otherwise follow pediatric infections, and in turn, altered intrinsic features of the immune system. Evidences that Treg differentiation, survival and expansion depend on IL-2 produced by effector cells^28^,^52^, together with their ability to directly respond to TLR ligands^53^, and therefore bacterial, viral and extra-cellular matrix component, provided a cellular and molecular basis to the hygiene hypothesis. This theory predicts overall lower number or decreased robustness of Treg in “cleaner environment”, a situation somewhat mimicked by transient and mild depletion of Treg as we performed here. In keeping with environmental and life-style changes, recent findings indicate that beneficial components of our microbiota promote Treg expansion and differentiation^54^, and the now routine practices of administrating frequent boost of antibiotics may have increased our susceptibility to autoimmunity.

## Material and Methods

### Mice and Antigens

Mouse strains used in this study were NOD/LtJ (NOD/ShiLtJ), C57BL/6 and BALB/c purchased from The Jackson Laboratory (Bar Harbor, ME). NOD-IFNγ^−/−^ (NOD.129S7(B6)-IFN-γ^tm1Ts^) mice were kindly provided by Dr. Diane Mathis and Christophe Benoist (The Jackson Laboratory). All animals were housed and maintained under SPF conditionsin the Animal Facilities of the Facultad de Ciencias Químicas, Universidad Nacional de Córdoba or the Instituto Gulbenkian de Ciência, and used at the age of 6–8 wk. All animal experiments were approved by and conducted in accordance with guidelines of the Committee for Animal Care and Use of the Facultad de Ciencias Químicas, Universidad Nacional de Córdoba and the ethics committee of the Instituto Gulbenkian de Ciência in strict accordance with the recommendation of the Guide to the Care and Use of Experimental Animals published by the Canadian Council on Animal Care (OLAW Assurance number A5802-01) and the Portuguese State Veterinary division (DGV).

The preparation of a mixture of prostate antigens (PAg) and the purification of Prostatein, or prostate steroid-binding protein (PSBP), were performed as previously described^55^. The purity of the PSBP preparation was >95% as evaluated by Western blot and was LPS-free as tested by Gel clot 0.03 endotoxin units/ml sensitivity (Charles River, Laboratories International, Wilmington, NY, USA).

### Antibodies

Commercially available antibodies used in different experiments performed and their respective manufacturer were as follows: anti-CD4 (RM4-5), anti-CD11b (M1/70), anti-CD3 (145-2C11), anti-GR1 (RB6-8C5); anti-CD44 (IM7), anti-CD62L (MEL-14), anti-IFNγ (XMG1.2), anti-IL-17A (TC11-18H10), and IgG isotype controls were purchased from BD Biosciences (San Diego, CA, USA). Anti-CD45 (30-F11), Anti-CD8a (53–6.7), anti-IL-10 (JES5-16E3), anti-IL-17A (eBIO17B7), anti-CD25 (PC61 and 7D4); anti-Foxp3 (FJK-16s) and Foxp3 Staining Buffer were purchased from eBioscience (San Diego, CA, USA). Anti-CD45 (30-F11), anti-CD11b (M1/70), anti-CCR6 (29-2L17), anti-CXCR3 (CXCR3-173), anti-CCR4 (2G12) and anti-CCR5 (HM-CCR5) were purchased from BioLegend (San Diego, CA, USA).

### EAP induction and histological prostatitis score

Six- to 8-week-old male NOD, C57BL/6, BALB/c or NOD-IFNγ^−/−^ mice were subcutaneously immunized in the hind footpad and in the base of the tail with PAg (300 μg/mouse) or saline solution emulsified in CFA (Sigma-Aldrich, St. Louis, MO, USA) in a total volume of 150 μl/mouse. Mice received immunizations at days 0 and 15, and then were sacrificed at day 24 of the experimental schedule^9^,^24^. The severity of EAP was assessed by determining the histological score, which was analyzed in a double-blind manner and computed for individual glands by summing the pathologic grade of each prostate tissue section and dividing by the total number of sections examined. The degree of inflammation was assessed using a score of 0 to 4:0, no inflammation; 1, mild but definite perivascular cuffing with mononuclear cells; 2, moderate perivascular cuffing with mononuclear cells; 3, marked perivascular cuffing, hemorrhage with some parenchymal inflammatory cells; 4, marked perivascular cuffing, hemorrhage and numerous mononuclear and mast cells in the parenchyma, in 5-mm-thick prostate tissue sections of each organ per animal that were processed by conventional hematoxylin and eosin staining. Slides were visualized in a microscope Nikon TE 2000U (Nikon, Osaka, Japan).

### *In vivo* CD25^+^ cell depletion

NOD, C57BL/6, BALB/c and NOD-IFNγ^−/−^ mice were injected intravenously (i.v.) with 500 μg of anti-CD25Ab (clone PC61). Control mice received the same amount of rat IgG Ab (clone YCATE) produced at the Instituto Gulbenkian of Ciência. Depletion was evaluated using anti-CD25 Ab (clone 7D4) and Foxp3 staining. Mice from all strains received anti-CD25 or IgG control Ab 1 day before starting the EAP immunization schedule as previously described^28^.

### *In vivo* treatment with recombinant IFNγ

NOD–IFNγ^−/−^ were immunized to induce EAP and then received intraperitoneal (i.p.) injections with 10^4^ U/day/mouse of rIFNγ (Peprotech, D.F., Mexico) resuspended in 100 μL of 0.1% BSA/PBS. Recombinant IFNγ was administrated on days 16, 18, 20, and 22 of the EAP experimental schedule. In some experiments, mice were injected with anti-CD25 or IgG control Ab on the day −1, then started the immunization protocol and were treated with rIFNγ on days 16, 18, 20 and 22 of the EAP experimental schedule.

### Prostate antigen specific antibodies in serum

Prostate steroid-binding protein–specific IgG1, IgG2a-c serum levels were titrated by conventional ELISA using specific detection antibodies in multiwell plates (Corning, Costar, Cambridge, MA). Plates were precoated with 100 μL per well of PSBP (20 mg/mL) in 0.05 M carbonate buffer of pH9.6. After overnight incubation at 4 °C, microwells were washed twice and blocked with 3% BSA (Sigma-Aldrich) in PBS for 2 hours at 37 °C, rinsed with PBS-Tween 20 at 0.05%, and then filled with 100 μL of serum (obtained after cardiac puncture) serial dilutions (starting at 1/50) for 1 hour at 37 °C. To detect specific IgG1, IgG2a-c plates were again washed and incubated with HRP conjugated rat anti-mouse IgG1 or anti-mouse IgG2a-c (BD Biosciences) for 1 hour at 37 °C. Plates were thoroughly washed, and the reaction was developed with BD OptEIA TMB Substrate Reagent Set (BD Biosciences). Absorbance was measured at 450 nm in a microplate reader (Bio-Rad Laboratories, Hercules, CA). Serum reactivity was expressed in titer inversed values.

### Cell culture

Single mononuclear cell suspensions were prepared in HBSS (Sigma-Aldrich) from spleen and pooled draining lymph nodes of individual mice by Ficoll-Paque PREMIUM 1.084 (GE Healthcare Bio-Sciences AB, Uppsala, Sweden) centrifugation gradients. Live cells were counted by Trypan blue exclusion, resuspended in RPMI 1640-GlutaMAX medium (Life Technologies, Carlsbad, CA, USA) supplemented with 1% penicillin/streptomycin (Life Technologies), 50 mM 2-ME (Life Technologies), and 10% FCS (Life Technologies), and cultured in the presence of PSBP (20 μg/ml) or medium alone. Plates were incubated at 37 °C in a water-saturated 5% CO_2_ atmosphere for 72 hours. After that, cells were processed for surface or/and intracellular cytokine staining and analyzed by FACS. Supernatants were frozen at −80 °C for cytokine quantifications.

### Cytokine quantification

Cytokine secretion of cell suspensions was assessed after antigen stimulation at a cell density of 1.5 × 10^6^ cell/ml. Cell suspension from spleen or prostate draining lymph nodes were stimulated during 72 h with PSBP (20 μg/ml). IFNγ, IL-17A and IL-10 concentrations in culture supernatants were measured by ELISA using paired Abs or specific kits (eBiosciences for IL-17A, and BD Biosciences for IFN-γ and IL-10) according to standard protocols and following manufacturer’s instructions.

### Flow cytometry

Freshly isolated mononuclear cells from spleen or LN were stained for surface markers and intracellular cytokines as previously described^9^,^24^. To determine the expression of chemokine receptors, cells were stimulated *in vitro* with PSBP and then staining was performed. In other experiments, after *in vitro* stimulation with PSBP (20 μg/ml) for 72 h, cells were incubated for 5 h. with 50 nM PMA and 0.5 μg/ml ionomycin (Sigma-Aldrich) in the presence of Golgi Stop (BD Biosciences). Cell-surface staining of different molecules and chemokines receptors was performed followed by intracellular staining of different cytokines using the BD CytoFix/Cyto Perm and Perm/Wash kit (BD Biosciences) according to the manufacturer’s instructions. Cells were incubated with APC-labeled antibodies to IL-17A (eBiosciences), or CCR6 (BioLegend), and with PE-labeled antibodies to IFNγ (BD Biosciences) or CXCR3 (BioLegend). Cells were acquired on FACS Canto II or FACS Calibur flow cytometer (BD Bioscience) and analyzed using FlowJo software (Tree Star, Ashland, OR, USA).

### Analysis of prostate infiltrating leucocytes

Prostate infiltrating leucocytes analysis was performed as previously described^9^,^24^. Freshly harvested prostate tissue samples were mechanically disrupted and enzymatically digested in RPMI 1640 medium containing 1 mg/ml collagenase D (Roche, Basilea, Switzerland) and DNase I (Sigma-Aldrich) for 45 min at 37 °C. After digestion, suspensions were filtered through 75- and 40-μm cell strainers (BD Biosciences) and single-cell suspensions were washed twice in 10% FBS, 2 mM EDTA, and 50 mM 2-ME supplemented RPMI 1640 medium. Live lymphocyte counts were deduced from the acquisition of a fixed number of 10-μm latex beads (Beckman Coulter, Brea, CA, USA) mixed with a known volume of unstained cell suspension in propidium iodide (BD Biosciences). Analyses were performed allowing the exclusion of dead cells (propidium iodide positive) inside the indicated gates. After that, cells were stained with different antibodies for flow cytometry analysis. Cells were acquired using a FACS Canto II or FACS Calibur (BD Bioscience), and data were analyzed using FlowJo software (Tree Star, Ashland, OR).

### Immunohistochemistry assays

Formalin-fixed and paraffin-embedded prostate sections were dewaxed in xylene, rehydrated, treated with Target Retrieval Solution (Dako Cytomation, Glostrup, Denmark) at 95 °C for 30 min, and blocked with FC blocking solution (BD Bioscience). Endogenous peroxidase activity was blocked with blocking buffer (Dako Cytomation). Then slides were incubated overnight at 4 °C in blocking buffer containing rabbit anti-CD45 (clone 30-F11) Ab. Slides were washed four times in 10 mM PBS, 0.1% Tween 20, and incubated with anti-rabbit HRP (BD Bioscience) secondary Abs for 2 h at room temperature. Colorimetric detection was performed by using Detection Kit (BD Bioscience) and counter stained with hematoxylin.

### Statistics

Statistical analysis was performed using one-way or two-way ANOVA with Bonferroni post hoc test analysis. Mean ± SEM are represented in the graphs. Statistical tests were performed using the Graph Pad Prism 5.0 software. The *p* value *p < 0.05 was considered significant in all analyses.

## Additional Information

**How to cite this article**: Breser, M. L. *et al.* Regulatory T cells control strain specific resistance to Experimental Autoimmune Prostatitis. *Sci. Rep.* **6**, 33097; doi: 10.1038/srep33097 (2016).

## Supplementary Material

Supplementary Information

## References

[b1] Lucas C. L. & Lenardo M. J. Identifying genetic determinants of autoimmunity and immune dysregulation. Curr.Opin.Immunol. 37, 28–33 (2015). 26433354 10.1016/j.coi.2015.09.001 PMC5583726

[b2] Bluestone J. A., Bour-Jordan H., Cheng M. & Anderson M. T cells in the control of organ-specific autoimmunity. J. Clin. Invest. 125, 2250–2260 (2015). 25985270 10.1172/JCI78089 PMC4497750

[b3] Forrester J. V., Klaska I. P., Yu T. & Kuffova L. Uveitis in mouse and man. Int. Rev. Immunol. 32, 76–96 (2013). 23360160 10.3109/08830185.2012.747524

[b4] Morris G. P., Brown N. K. & Kong Y. C. Naturally-existing CD4(+)CD25(+)Foxp3(+) regulatory T cells are required for tolerance to experimental autoinmune thyroiditis induced by either exogenous or endogenous autoantigen. J. Autoimmun. 33, 68–76 (2009). 19375891 10.1016/j.jaut.2009.03.010 PMC2706097

[b5] Allenbach Y. *et al.* Role of regulatory T cells in a new mouse model of experimental autoimmune myositis. Am. J. Pathol. 174, 989–998 (2009). 19218348 10.2353/ajpath.2009.080422 PMC2665758

[b6] Seki N. *et al.* Type II collagen-induced murine arthritis: induction of arthritis depends on antigen-presenting cell function as well as susceptibility of host to an anti collagen immune response. J. Immunol. 148, 3093–3099 (1992). 1578134

[b7] Bernard C. C. *et al.* Myelin oligodendrocyte glycoprotein: a novel candidate autoantigen in multiple sclerosis. J. Mol. Med. 75, 77–88 (1997). 9083925 10.1007/s001090050092

[b8] Motrich R. D., Maccioni M., Riera C. M. & Rivero V. E. Autoimmune prostatitis: state of the art. Scand. J. Immunol. 66, 217–227 (2007). 17635799 10.1111/j.1365-3083.2007.01971.x

[b9] Breser M. L., Motrich R. D., Sanchez L. R., Mackern-Oberti J. P. & Rivero V. E. Expression of CXCR3 on specific T cells is essential for homing to the prostate gland in an experimental model of chronic prostatitis/chronic pelvic pain syndrome. J. Immunol. 190, 3121–3133 (2013). 23455510 10.4049/jimmunol.1202482

[b10] Tsai S. & Santamaria P. MHC Class II Polymorphisms, Autoreactive T-Cells, and Autoimmunity. Front. Immunol. 4(321), 1–7 (2013). 24133494 10.3389/fimmu.2013.00321 PMC3794362

[b11] Siggs O. M., Makaroff L. E. & Liston A. The why and how of thymocyte negative selection. Curr. Opin. Immunol. 8, 175–183 (2006). 16459069 10.1016/j.coi.2006.01.001

[b12] Danke N. A., Koelle D. M., Yee C., Beheray S.& Kwok W. W. Autoreactive T cells in healthy individuals. J. Immunol. 172, 5967–5972 (2004). 15128778 10.4049/jimmunol.172.10.5967

[b13] Reiner S. L., Wang Z. E., Hatam F., Scott P. & Locksley R. M. TH1 and TH2 cell antigen receptors in experimental leishmaniasis. Science. 259, 1457–1460 (1993). 8451641 10.1126/science.8451641

[b14] Rosenblum M. D. *et al.* Response to self antigen imprints regulatory memory in tissues. Nature. 480, 538–542 (2011). 22121024 10.1038/nature10664 PMC3263357

[b15] Campbell D. J. Control of Regulatory T Cell Migration, Function, and Homeostasis. J. Immunol. 195, 2507–2513 (2015). 26342103 10.4049/jimmunol.1500801 PMC4778549

[b16] Fontenot J. D., Gavin M. A. & Rudensky A. Y. Foxp3 programs the development and function of CD4+ CD25+ regulatory T cells. Nat. Immunol. 4, 330–336 (2003). 12612578 10.1038/ni904

[b17] Barzaghi F., Passerini L. & Bacchetta R. Immunedysregulation, polyendocrinopathy, enteropathy, X-linked syndrome: a paradigm of immunodeficiency with autoimmunity. Front. Immunol. 3(211), 1–25 (2012). 23060872 10.3389/fimmu.2012.00211 PMC3459184

[b18] Pandiyan P. & Zhu J. Origin and functions of pro-inflammatory cytokine producing Foxp3+ regulatory T cells. Cytokine. 76, 13–24 (2015). 26165923 10.1016/j.cyto.2015.07.005 PMC4969074

[b19] Schmidt A., Oberle N. & Krammer P. H. Molecular mechanisms of Treg-mediated T cell suppression. Front. Immunol. 21, 3–51 (2012). 10.3389/fimmu.2012.00051 PMC3341960 22566933

[b20] Arpaia N. *et al.* A Distinct Function of Regulatory T Cells in Tissue Protection. Cell. 162, 1078–1089 (2015). 26317471 10.1016/j.cell.2015.08.021 PMC4603556

[b21] Rivero V. E., Motrich R. D., Maccioni M. & Riera C. M. Autoimmune etiology in chronic prostatitis syndrome: an advance in the understanding of this pathology. Crit. Rev. Immunol. 27, 33–46 (2007). 17430095 10.1615/critrevimmunol.v27.i1.30

[b22] Schaeffer A. J. Clinical practice. Chronic prostatitis and the chronic pelvic pain syndrome. N. Engl. J. Med. 355, 1690–1698 (2006). 17050893 10.1056/NEJMcp060423

[b23] Motrich R. D. *et al.* Presence of INF gamma-secreting lymphocytes specific to prostate antigens in a group of chronic prostatitis patients. Clin. Immunol. 116, 149–157 (2005). 15993362 10.1016/j.clim.2005.03.011

[b24] Motrich R. D. *et al.* IL-17 is not essential for inflammation and chronic pelvic pain development in an experimental model of chronic prostatitis/chronic pelvic pain syndrome. Pain. 157, 585–597 (2016). 26882345 10.1097/j.pain.0000000000000405

[b25] Keetch D. W., Humphrey P. & Ratliff T. L. Development of a mouse model for nonbacterial prostatitis. J. Urol. 152, 247–250 (1994). 8201676 10.1016/s0022-5347(17)32871-9

[b26] Chen X., Oppenheim J. J. & Howard O. M. BALB/c mice have more CD4+ CD25+ T regulatory cells and show greater susceptibility to suppression of their CD4+ CD25- responder T cells than C57BL/6 mice. J Leukoc Biol. 78, 114–21 (2005). 15845645 10.1189/jlb.0604341

[b27] Tucker C. F. *et al.* Decreased frequencies of CD4+ CD25+ Foxp3+ cells and the potent CD103+ subset in peripheral lymph nodes correlate with autoinmune disease predisposition in some strains of mice. Autoimmunity. 44, 453–64 (2011). 21604973 10.3109/08916934.2011.568553

[b28] Zelenay S. *et al.* Foxp3+ CD25− CD4 T cells constitute a reservoir of committed regulatory cells that regain CD25 expression upon homeostatic expansion. Proc. Natl. Acad. Sci. USA. 102, 4091–4096 (2005). 15753306 10.1073/pnas.0408679102 PMC554795

[b29] Zelenay S. & Demengeot J. Comment on “Cutting edge: anti-CD25 monoclonal antibody injection results in the functional inactivation, not depletion, of CD4+ CD25+ T regulatory cells”. J. Immunol. 177, 2036–2037 (2006). 16887957 10.4049/jimmunol.177.4.2036-a

[b30] Maccioni M., Rivero V. E. & Riera C. M. Prostatein (or rat prostatic steroid binding protein) is a major autoantigen in experimental autoimmune prostatitis. Clin. Exp. Immunol. 112, 159–165 (1998). 9649176 10.1046/j.1365-2249.1998.00588.x PMC1904968

[b31] Liu K. J. *et al.* Identification of rat prostatic steroid-binding protein as a target antigen of experimental autoimmune prostatitis: implications for prostate cancer therapy. J. Immunol. 159, 472–480 (1997). 9200488

[b32] Sallusto F. & Lanzavecchia A. Heterogeneity of CD4+ memory T cells: functional modules for tailored immunity. Eur. J. Immunol. 39, 2076–2082 (2009). 19672903 10.1002/eji.200939722

[b33] Sakaguchi S. Regulatory T cells: history and perspective. Methods Mol. Biol. 707, 3–17 (2010). 21287325 10.1007/978-1-61737-979-6_1

[b34] Amu S., Gjertsson I. & Brisslert M. Functional characterization of murine CD25 expressing B cells. Scand. J. Immunol. 71, 275–282 (2010). 20384871 10.1111/j.1365-3083.2010.02380.x

[b35] Liang D. *et al.* Role of CD25+ dendritic cells in the generation of Th17 autoreactive T cells in autoimmune experimental uveitis. J Immunol. 188, 5785–5791 (2012). 22539790 10.4049/jimmunol.1200109 PMC3358586

[b36] J. D. 1 Fontenot, Gavin M. A. & Rudensky A. Y. Foxp3 programs the development and function of CD4+ CD25+ regulatory T cells. Nat Immunol. 4, 330–6 (2003). 12612578 10.1038/ni904

[b37] Kim J. M., Rasmussen J. P. & Rudensky A. Y. Regulatory T cells prevent catastrophic autoimmunity throughout the lifespan of mice. Nat. Immunol. 8, 191–197 (2006) 17136045 10.1038/ni1428

[b38] Lahl K. *et al.* Selective depletion of Foxp3+ regulatory T cells induces a scurfy-like disease. J. Exp. Med. 204, 57–63 (2007). 17200412 10.1084/jem.20061852 PMC2118432

[b39] Meyer zu Hörste G. *et al.* Active immunization induces toxicity of diphtheria toxin in diphtheria resistant mice. Implications for neuroinflammatory models. J. Imm. Methods. 354, 80–84 (2010). 10.1016/j.jim.2010.01.012 20138048

[b40] Kohm A. P. *et al.* Cutting Edge: Anti-CD25 monoclonal antibody injection results in the functional inactivation, not depletion, of CD4+ CD25+ T regulatory cells. J.Immunol. 176, 3301–3305 (2006). 16517695 10.4049/jimmunol.176.6.3301

[b41] Goebel J., Stevens E., Forrest K. & Roszman T. L. Daclizumab (Zenapax) inhibits early interleukin-2 receptor signal transduction events. Transpl. Immunol. 8, 153–159 (2000). 11147695 10.1016/s0966-3274(00)00021-6

[b42] Fecci P. E. *et al.* Systemic anti-CD25 monoclonal antibody administration safely enhances immunity in murine glioma without eliminating regulatory T cells. Clin. Cancer Res. 12, 4294–4305 (2006). 16857805 10.1158/1078-0432.CCR-06-0053

[b43] Taguchi O., Kojima A. & Nishizuka Y. Experimental autoimmune prostatitis after neonatal thymectomy in the mouse. Clin. Exp. Immunol. 60, 123–129 (1985). 4006298 PMC1577003

[b44] Wheeler K. M., Samy E. T. & Tung K. K. Cutting edge: normal regional lymph node enrichment of antigen-specific regulatory T cells with autoimmune disease-suppressive capacity. J. Immunol. 183, 7635–7638 (2009). 19923458 10.4049/jimmunol.0804251 PMC2872190

[b45] Felonato M. *et al.* Anti-CD25 treatment depletes Treg cells and decreases disease severity in susceptible and resistant mice infected with Paracoccidioides brasiliensis. PLoS One. 7, e51071 (2012). 23226464 10.1371/journal.pone.0051071 PMC3511355

[b46] Nyström S. N. *et al.* Transient Treg-cell depletion in adult mice results in persistent self-reactive CD4(+) T-cell responses. Eur. J. Immunol. 44, 3621–3631 (2014). 25231532 10.1002/eji.201344432

[b47] Coutinho A. *et al.* Thymic commitment of regulatory T cells is a pathway of TCR-dependent selection that isolates repertoires undergoing positive or negative selection. Current Topics in Microbiology and Immunology. 293, 43–71 (2005). 15981475 10.1007/3-540-27702-1_3

[b48] Kieback E. *et al.* Thymus-Derived Regulatory T Cells Are Positively Selected on Natural Self-Antigen through Cognate Interactions of High Functional Avidity. Immunity. 44, 1114–1126 (2016). 27192577 10.1016/j.immuni.2016.04.018

[b49] Malchow S., Leventhal D. S., Lee V., Nishi S., Socci N. D. & Savage P. A. Aire Enforces Immune Tolerance by Directing Autoreactive T Cells into the Regulatory T Cell Lineage. Immunity. 44, 1102–1113 (2016). 27130899 10.1016/j.immuni.2016.02.009 PMC4871732

[b50] Bromley S. K., Mempel T. R. & Luster A. D. Orchestrating the orchestrators: chemokines in control of T cell traffic. Nat. Immunol. 9, 970–980 (2008). 18711434 10.1038/ni.f.213

[b51] Okada H., Kuhn C., Feillet H. & Bach J. F. The ‘hygiene hypothesis’ for autoimmune and allergic diseases: an update. Clin. Exp. Immunol. 160, 1–9 (2010). 20415844 10.1111/j.1365-2249.2010.04139.x PMC2841828

[b52] Duarte J. H., Zelenay S., Bergman M. L., Martins A. C. & Demengeot J. Natural Treg cells spontaneously differentiate into pathogenic helper cells in lymphopenic conditions. Eur. J. Immunol. 39, 948–955 (2009). 19291701 10.1002/eji.200839196

[b53] Caramalho I. *et al.* Regulatory T cells selectively express toll-like receptors and are activated by lipopolysaccharide. J. Exp. Med. 197, 403–411 (2003). 12591899 10.1084/jem.20021633 PMC2193858

[b54] Arpaia N. *et al.* Metabolites produced by commensal bacteria promote peripheral regulatory T-cell generation. Nature. 504, 451–455 (2013). 24226773 10.1038/nature12726 PMC3869884

[b55] Motrich R. D. *et al.* Pathogenic consequences in semen quality of an autoimmune response against the prostate gland: from animal models to human disease. J. Immunol. 177, 957–967 (2006). 16818751 10.4049/jimmunol.177.2.957

