# Supplementary material for: Regulatory T cells control strain specific resistance to Experimental Autoimmune Prostatitis
Source: Sci Rep. 2016 Sep 14;6:33097. doi: 10.1038/srep33097 (PMC5022010; doi:10.1038/srep33097)
Supplement: Supplementary Information [file srep33097-s1.pdf]

Supplementary information

Regulatory T cells control strain specific resistance to Experimental Autoimmune Prostatitis

Maria L. Breser, Andreia C. Lino, Ruben D. Motrich, Gloria J. Godoy, Jocelyne Demengeot, Virginia E. Rivero.

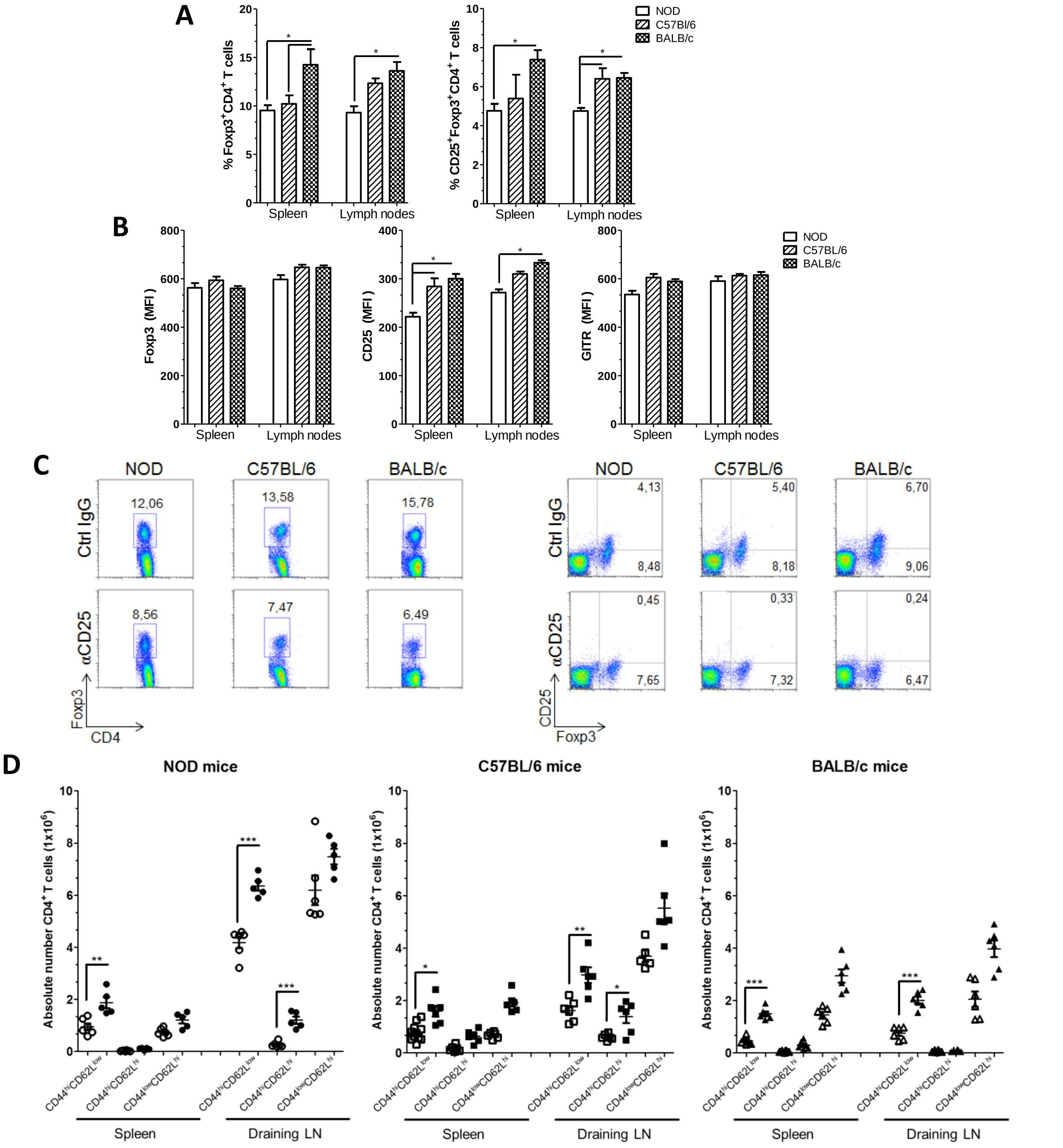

Supplementary figure 1. Frequency and phenotype of Treg in NOD, C57BL/6 and BALB/c animals at steady state. Spleen and Lymph nodes were analyzed by FACS to define the frequency of $Foxp3^+$ or $CD25^+Foxp3^+$ inside a $CD4^+$ gate (A), and the Mean fluorescence intensity (MIF) of Foxp3, CD25 and GITR inside a $Foxp3^+$ gate (B). (C) representative FACS dot plots assessing the frequency of $Foxp3^+$ or $CD25^+Foxp3^+$ inside a $CD4^+$ gate in the 3 strains upon immunization and prior administration of Control Ab or anti-CD25 Ab, as depicted in Fig. 1A. Compilation of all data is presented in Fig 1B. (D) The analysis presented in Fig. 1D left panel, was extended to the Spleen in addition to Lymph nodes. Plotted are the absolute number of $CD44^{hi}$ $CD62L^{low}$, $CD44^{hi}$ $CD62L^{hi}$, $CD44^{low}$ $CD62L^{hi}$ T cells recovered in each strain upon immunization and prior administration of Control IgG Ab or anti-CD25 Ab.

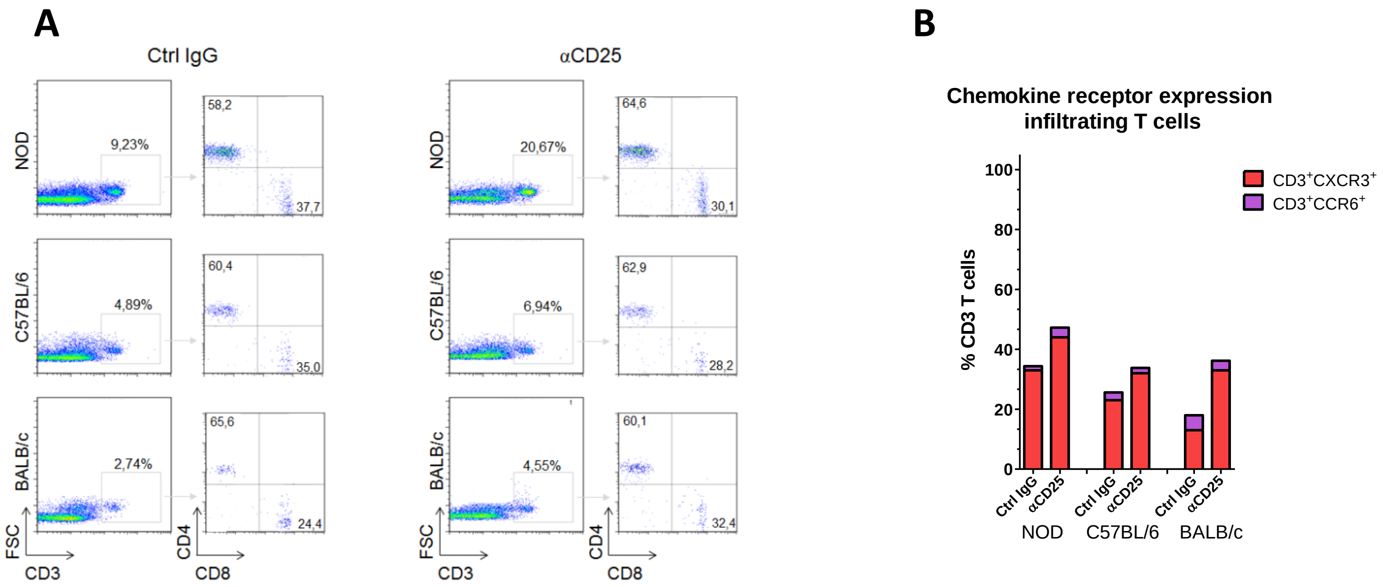

Supplementary figure 2. Characterization of prostate infiltrate. (A) FACS analysis of the prostate infiltrates presented in Fig. 3C was also processed to determine the frequency of CD3$^+$ cells inside a CD45$^+$ gate and the frequency of CD4 and CD8 T cells inside a CD3$^+$ gate. Shown are representative dot plots for each experimental group. (B) Analysis of CXCR3$^+$ and CCR6$^+$ cell in the prostate infiltrates presented as absolute numbers in Fig. 3F were also treated as relative values. Shown are the frequency of CXCR3$^+$ and CCR6$^+$ cells inside a CD3 gate, compiled for each experimental group.
